# Maternal and Early Postnatal Diet Supplemented with Conjugated Linoleic Acid Isomers Affect Lipid Profile in Hearts of Offspring Rats with Mammary Tumors

**DOI:** 10.3390/ani10030464

**Published:** 2020-03-11

**Authors:** Małgorzata Białek, Agnieszka Białek, Marian Czauderna

**Affiliations:** 1 The Kielanowski Institute of Animal Physiology and Nutrition, Polish Academy of Sciences, Instytucka 3, 05-110 Jabłonna, Poland; m.czauderna@ifzz.pl; 2 Department of Animal Improvement and Nutrigenomics, Institute of Genetics and Animal Breeding, Polish Academy of Sciences, Postępu 36A Jastrzębiec, 05-552 Magdalenka, Poland

**Keywords:** nutritional programming, maternal diet, postnatal diet, conjugated linoleic acid, cancer, heart, rats

## Abstract

**Simple Summary:**

Proper feeding during pregnancy and breastfeeding plays an important role in ensuring the health of offspring in childhood as well as in adulthood. The idea of the “developmental origin of health and disease” (DOHaD) assumes that stimuli acting during fetal life cause long-term developmental and physiological changes in the main organs and influence the risk of many diseases. Our research, connected with DOHaD, made an attempt to evaluate the impact of the diet modification of mothers through the addition of conjugated linoleic acid isomers (CLA) on the lipid profile, fatty acid profile and the oxidative stress in the hearts of offspring with breast cancer. Earlier studies utilizing CLA in DOHaD concerned their impact on reducing the incidence of cancer. Considering reports on the co-occurrence of cardiovascular diseases (CVD) in oncological patients as a result of tumor development and also after chemotherapy and radiotherapy, further research is needed to assess the potential protective effect of CLA isomers in CVD. For this purpose, the content of selected CVD biomarkers was determined using modern analytical techniques. Our research has tried to explain the physiological role of CLA and its mechanism of action in the context of maintaining good health and in the prevention of non-communicable diseases.

**Abstract:**

Linking the early life environment with later health status is known as “developmental programming”. This study aimed to assess whether the introduction of conjugated linoleic acids (CLAs) into the maternal diet affects the content fatty acids (FAs), conjugated FAs (CFAs), cholesterol, oxysterols, malondialdehyde (MDA) and tocopherols in the hearts of their female offspring treated with 7,12-dimethylbenz[a]anthracene and if offspring supplementation enhanced the effect of maternal supplementation. FA, cholesterol and oxysterol contents were determined by gas chromatography-mass spectrometry, while contents of CFAs and MDA were determined by high-performance liquid chromatography (HPLC) with photodiode detection. The supplementation of mothers with CLAs significantly decreased the amount of atherogenic saturated FAs and enhanced the level of eicosapentaenoic FA in the hearts of offspring. Continuous progeny supplementation decreased the content of arachidonic acid in hearts. Supplementation of the maternal diet with CLAs and its continuation during the postnatal period increased the ratio of hypo to hypercholesterolemic FAs. Significantly fewer oxysterols were detected in the hearts of progeny of dams fed with CLAs as compared to the offspring of mothers receiving safflower oil. Both fetal and postnatal CLA intake significantly reduced 7β-hydroxycholesterol accumulation. It can be concluded that CLA supplementation during the fetal and postnatal period may be an effective method of maintaining the cardiac health status of newborns.

## 1. Introduction

Over the last two decades, a significant change in the global pattern of diseases has been observed, resulting from communicable and perinatal causes to mainly noncommunicable diseases (NCD) [1]. NCD, also called chronic diseases, are long-term disorders with generally slow development. Among the main types of NCDs, the most prevalent are cardiovascular diseases (CVD) and cancer, which are also responsible for a substantial decrease of lifespan and the majority of deaths globally [2,3]. As estimated by the World Health Organization (WHO) nearly 18 million people die from CVD yearly (31% of all deaths) [2]. According to the International Agency for Research on Cancer (IARC), breast cancer is the second most common type of cancer (preceded only by lung cancer) and the most frequent cause of death for women worldwide [3]. Currently, there is an increasing amount of evidence that cancer and CVD share not only the risk factors but also the mechanism of pathogenicity [4,5]. This is confirmed mainly in the case of breast cancer by the increased cardiac risk in patients receiving chemotherapy and radiotherapy as well as in cancer survivors [6,7,8,9,10]. It is also plausible that cancer itself may deleteriously affect heart function, irrespective of exposure to anticancerogenic therapies. The results of the research by Khattar’s team indicated that the growing tumor itself may cause direct injury to the heart of patients with cancer who have not yet been treated [11]. Taking into account both the tremendous health and financial costs of CVD and cancer [12,13], searching for the fundamental biological mechanisms of these chronic diseases, and thus effective prevention strategies, should be of utmost importance. 

Strong social beliefs that NCD is mainly associated with genetic abilities and unhealthy lifestyle are currently confronted with the growing amount of evidence confirming the early origins of chronic diseases [13]. The idea of linking early life environmental factors with later health status, developed in the 1980s by David Baker, has recently become known as “developmental origins of health and disease” (DOHaD) [14] or “developmental programming” [15]. The definition of “programming” implies that exposure to a particular stimulus acting during critical phases of development (embryonic and fetal life) introduces long-term developmental, physiological and metabolic changes in the main tissues and organs [16,17]. During gestation, diet is considered to be the most important factor affecting fetal development and metabolism [18]. The results of both human epidemiological studies and animal experiments confirmed that nutritional exposure during critical periods of development affects gene expression and thus affects metabolism [19,20,21,22]. The nutritional influence on the developmental establishment of epigenetic regulatory mechanisms connects early life nutrition with chronic disease susceptibility in adulthood, although the explanation of this phenomena is still rudimentary [15]. 

To date, a plethora of experiments have focused mainly on the under and over-nutrition of mothers (e.g., a low protein diet [23], high fat diet [24], caloric restrictions [25], and obesity [26]). Recent studies have shown that bioactive compounds introduced into the maternal diet may significantly influence the composition and/or functioning of certain tissues and organs [27,28,29]. One of the most important nutrients required for fetal growth and development is fatty acids (FAs). As the fetus has a limited ability to metabolize derived FAs, these essential compounds should be supplied to the mother in sufficient amounts for fetal development [30]. As the activity of desaturases in the placenta is very low, there is no biosynthesis of long-chained polyunsaturated FA (PUFA) such as eicosapentaenoic *(c*5*c*8*c*11*c*14*c*17C20:5; EPA), docosahexaenoic (*c*4*c*7*c*10*c*13*c*16*c*19C22:6; DHA) and arachidonic (*c*5*c*8*c*11*c*14C20:4; AA) acids from their precursors, α-linolenic (*c*9*c*12*c*15C18:3; ALA) and linoleic (*c*9*c*12C18:2; LA) acids, and thus, the only source of these FAs relevant for organogenesis and neural system development is maternal plasma [31,32]. 

The influence of the maternal intake of selected groups of FAs on development and metabolic consequences for offspring has been intensively studied and reasonably proven. A high supply of saturated FA (SFA) or industrial trans isomers to mothers during critical stages of fetal development leads to adverse changes in liver and adipose tissue, which in turn are associated with an increased risk of insulin resistance or type 2 diabetes in the long-time health of offspring [18]. Adequate amounts and relative proportions of n-3 and n-6 PUFA in the mother’s diet during the perinatal period may modulate lipid status and metabolic pathways in various tissues of the progeny [33,34].

Among PUFAs, a distinct group of geometric and positional isomers with unsaturated bonds separated by one single bond—conjugated fatty acids (CFAs)—is distinguished. CFAs, due to their specific structure, exhibit unique chemical properties and physiological activity [35]. To date, the most extensively studied CFAs are conjugated linoleic acid (CLA), especially *c*9*t*11C18:2 and *t*10*c*12C18:2 isomers. These zoochemicals are known for their anti-cancerogenic, anti-atherogenic and immunostimulating properties [36,37,38,39,40]. Taking into account the fact that n-3 and n-6 FAs have been intensively investigated in “fetal programming” models, the examination of the role of other nutritionally important FAs, such as CLA, in ensuring the health of offspring by modifying the mother’s nutrition is justified. 

As it is possible to affect the cancer incidence among progeny by the modification of both maternal and postnatal diets by CLA incorporation [41], an assumption was made that certain CVD biomarkers may also be influenced by the supplementation of these CFAs. The exposure of mothers to PUFA within the perinatal period may modify the FA status of various organs, inter alia hearts, of their offspring and thus be involved in neonatal fat metabolism [18].

Therefore, the main aim of this preliminary study was to evaluate how the incorporation of CLA into the maternal diet influences FA and the metabolism of other lipid compounds in the hearts of their female offspring in whom cancerogenesis was chemically induced. A secondary goal was to find if the supplementation of the offspring’s diet in the early postnatal period can enhance the effect of the mothers’ supplementation.

## 2. Materials and Methods

### 2.1. Materials

#### 2.1.1. Dietary Ingredients

Laboratory fodder Labofeed H composed of 22.0% protein, 4.0% fat, 30.0% starch, 5.0% fibre, 6.5% minerals was purchased from “Morawski” Feed and Concentrates Production Plant (Kcynia, Poland). Commercially available Bio-C.L.A. dietary supplement in the form of gel capsules, containing an equimolar mixture of CLA isomers, was obtained free of charge from Pharma Nord (Warsaw, Poland). It was stored at room temperature according to the manufacturer’s recommendation until the oily filling was pressed out from the capsule and administered to the animals. Safflower oil (SAF oil), used as substrate for Bio-C.L.A. production, was donated by Pharma Nord (Warsaw, Poland). The composition of dietary ingredients is presented in Table 1. 

#### 2.1.2. Animal Experiment

This research and guiding principles in the care and use of laboratory animals were approved by the Second Local Ethical Committee on Animal Experiments (No. 34/2008) at the Medical University of Warsaw. According to the 3Rs (replacement, reduction and refinement) ethical principle, the design of the study and experimental techniques used through the analysis allowed us to minimize the number of animals while maintaining high statistical precision. There is no way to completely replace live animals with another research model in developmental programming experiments, especially in those concerning breast cancer development. Thus, Sprague–Dawley female rats and 7,12-dimethylbenz[a]anthracene (DMBA) as a cancerogenic agent were chosen as a model for breast cancer in humans, because of several similarities in physiology, metabolism and pathology.

**Figure 1:**
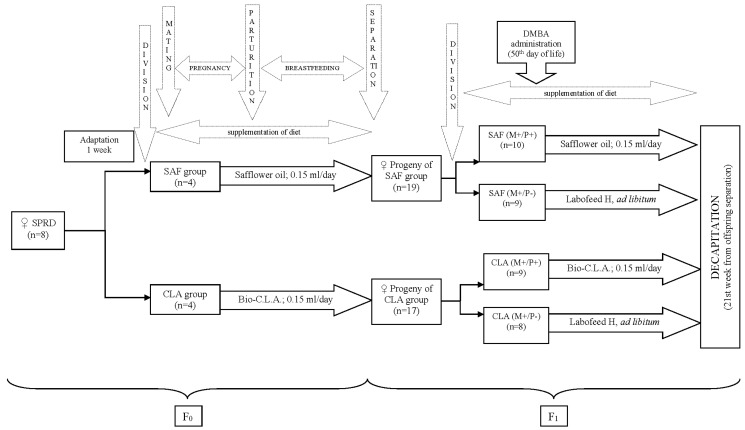
The experiment design.

Virgin female Sprague–Dawley rats (n = 8) were obtained from the Division of Experimental Animals, Department of General and Experimental Pathology (Medical University of Warsaw, Warsaw, Poland). They were housed in an animal room at 21 °C, in a 12 h light: 12 h dark cycle. Laboratory fodder was fed ad libitum during the entire experiment. After a 1 week adaptation, maternal animals were randomly assigned to two groups of four rats each: group SAF, supplemented with SAF oil, or group CLA, supplemented with Bio-C.L.A. Both supplements (0.15 mL per day per individual) were given intragastrically via gavage. After 10 days of supplementation, rats from SAF and CLA groups were mated with male Sprague–Dawley rats. Maternal diet supplementation was continued throughout gestation and lactation. To avoid the paternal effect, the male diet was not modified. On the 30th day of life, female progeny were separated from mothers and, within the supplementation groups, randomly divided into two subsequent groups. The numbers of animals included in each progeny group was strictly dependent on numbers of female pups in the litter. Diets of SAF(M+/P+) and CLA(M+/P+) progeny groups (n = 10 and n = 8, respectively) were supplemented with the same preparation that had been given to mothers (SAF oil and Bio-C.L.A., respectively), while the diets of SAF(M+/P−) and CLA(M+/P−) progeny groups (n = 9 and n = 8, respectively) were not supplemented. Dietary interventions in all offspring groups lasted for 21 weeks after separation from mothers. Each individual in every progeny group received—on the 50th day of life—a single dose of 80 mg/kg body weight intragastrically of chemical carcinogenic agent—DMBA (approx. 95%; Sigma-Aldrich, Saint Louis, MO, USA)—for the induction of mammary tumors. During the entire experiment, all rats (mothers and offspring) were under the constant care of an experienced veterinarian who daily monitored for specific signs of welfare disorders as well as weighing and palpating rats weekly for the detection of tumor appearances. All offspring animals were decapitated, exsanguinated and whole hearts were excised, weighed and stored in −80 °C for further analyses after 21 weeks. No biological samples were taken from maternal rats for analyses. A detailed scheme of the experiment is presented on Figure 1. 

SPRD—Sprague-Dawley rats; CLA—conjugated linoleic acids, DMBA—7,12-dimethylbenz[a]anthracene, CLA group—group of mothers receiving conjugated linoleic acids (Bio-C.L.A.) during pregnancy and breastfeeding, SAF group—group of mothers receiving safflower oil during pregnancy and breastfeeding, SAF(M+/P+)—group of offspring receiving safflower oil during fetal life, breastfeeding and after separation from mothers, SAF(M+/P−)—group of offspring receiving safflower oil only during fetal life and breastfeeding, CLA(M+/P+)—group of offspring receiving conjugated linoleic acids (Bio-C.L.A.) during fetal life, breastfeeding and after separation from mothers, CLA(M+/P−)—group of offspring receiving conjugated linoleic acids (Bio-C.L.A.) during fetal life and breastfeeding.

### 2.2. Methods

#### 2.2.1. CFA and FA Profile

Prior to the chromatographic analysis, hearts were subjected to alkaline hydrolysis. In order to determine CFAs (dienes, CD, and trienes, CT), Waters HPLC 625LC system (Milford, MA, USA), four ion-exchange columns loaded with silver ions (Chromspher Lipids 5 μm, 250 × 4.6 mm; Varian, The Netherlands) and a photodiode array detector (PDA) were used [42,43]. The FA profile was determined as methyl esters with the addition of nonadecanoic acid (C19:0) as the internal standard (IS). The analysis of methyl fatty acid (FAME) was performed with a SHIMADZU GC-MS-QP2010 Plus EI gas chromatograph (GC) equipped with quadruple mass selective detector Model 5973N (Tokyo, Japan) and fused silica capillary-column BPX70 (120 m × 0.25 mm × 0.25 μm; Phenomenex, Torrance, CA, USA) [43,44].

On the basis of the FA profile, indices attributed to the oxidative (peroxidability index, PI), atherogenic (index of atherogenicity, AI), thrombogenic (index of thrombogenicity, TI) and cholesterolemic (hypo/hypercholesterolemic FA ratio, HH) properties as well as to the isomerization rate (iso_LA and iso_ALA) were also calculated according to the following equations [43,45,46,47]:

(1) PI= (%monoenoic FA×0.025)+(% dienoic FA×1)+ (% trienoic FA×2)+(% tetraenoic FA×3)+ (% pentaenoic FA×4) +(% hexaenoic FA×5) 

(2) $$AI=\frac{C12:0+\left(4\times C14:0\right)+C16:0}{\sum MUFA+\sum n-6PUFA+\sum n-3PUFA}$$

(3) $$TI=\frac{C14:0+C16:0+C18:0}{\left(0.5\times \sum MUFA\right)+\left(0.5\times \sum n-6PUFA\right)+\left(3\times \sum n-3PUFA\right)+\left(\frac{\sum n-3PUFA}{\sum n-6PUFA}\right)}$$

(4) $$HH=\frac{OA+LA+AA+ALA+EPA+DPA+DHA}{C14:0+C16:0}$$

(5) $$iso\_LA=\;\frac{CD}{LA+CD}$$

(6) $$iso\_ALA=\;\frac{CT}{ALA+CT}$$

#### 2.2.2. Total Cholesterol and Oxysterol Content

Total cholesterol and its oxidized derivatives’ contents in rats’ hearts were assayed after alkaline saponification according to the method of Czauderna et al. [48] followed by silylation with BSTFA (*N*,*O*-Bis(trimethylsilyl)trifluoroacetamide, 99%; Sigma Aldrich, St. Louis, MO, USA). Briefly, the hexane layer obtained after saponification and extraction was evaporated under a stream of argon; next, 25 μL of BSTFA and 50 μL of pyridine were added to the dry residue. Afterwards, samples were heated at 80 °C for 40 min, cooled down at ambient temperature and then diluted in 225 μL of hexane. The resulting solution (1 μL) was then injected onto a capillary column (30 m × 0.25 mm × 0.25 μm film thickness, Rxi^®^-17SilMS, Restek, Bellefonte, PA, USA) coupled to the GC-TOFMS Pegasus^®^ BT (LECO Corporation, St. Joseph, MI, USA) chromatograph. The injector, interface and mass spectrometer temperatures were maintained at 300, 300, and 250 °C, respectively. The oven temperature was initially set at 200 °C for 4.6 min, then ramped to 290 °C at 5°/min and held for 12.4 min. Helium was used as a carrier gas at a constant flow (1.0 mL/min). For the identification and recoveries of analytes, standards of cholesterol and selected oxysterols (7α-hydroxycholesterol, 7AOH; 7β-hydroxycholesterol, 7BOH; cholesterol 5α,6α-epoxide, 5,6AE; cholesterol 5β,6β-epoxide, 5,6BE; 5α-cholestane-3,5,6-triol, triol; 7-ketocholesterol, 7K) (Sigma, St. Louis, MO, USA) were applied. Identification was made by the comparison of analytes’ retention times and mass spectra with these obtained for standards. As IS 5α-cholestane (Sigma, St. Louis, MO, USA) was used.

### 2.3. Malondialdehyde (MDA) Content

Heart samples were subjected to gentle alkaline saponification and derivatization with 2,4-dinitrophenylhydrazine (DNPH) followed by extraction with hexane, according to a previously published method [49]. A high-performance liquid chromatography (HPLC) UFLCXR system (SHIMADZU, Tokyo, Japan) equipped with a C18-column (Synergi 2.5 μm, Hydro-RP, 100 Å, 100 mm × 2 mm, Phenomenex, Torrance, CA, USA) and a PDA was used. The PDA operated in the UV range of 195–420 nm, and a CTO-20A column heater maintained the temperature at 40 °C. The 1,5-pentanedialdehyde solution was used as IS. A linear binary gradient of acetonitrile in water was used. Solvent A consisted of water–acetonitrile (95:5, *v/v*) and solvent B consisted of 100% acetonitrile. MDA identification was based on the retention time and absorption UV spectrum (λ_max_ = 306 nm) of the analytical standard (Sigma, St Louis, MO, USA).

#### 2.3.1. Tocopherol Content

Tocopherols (α-, γ-, δ-, acetate) in hearts were analyzed using a reversed-phase liquid chromatography with PDA (RP-HPLC-PDA) using a SHIMADZU UFLCXR chromatography system (Tokio, Japan) equipped with a C18-column (Kinetex 1.7 μm, 100Å, 150 × 2.1 mm, Phenomenex, Torrance, CA, USA) according to a previously described method [50]. 

#### 2.3.2. Statistical Analysis

The obtained results, presented as means ± standard deviation (SD), were elaborated with STATISTICA 13 software [51]. Effects of maternal diet (MD), offspring supplementation (OS) and interactions (MD × OS) were evaluated using a two-way ANOVA. The normality of the data distribution was checked by Shapiro–Wilk test. When interaction occurred (*p* ≤ 0.05), the significances of differences among groups were established by using a post hoc HSD Tukey test for uneven numbers for variables with a normal distribution or multiple comparison test for variables with a skewed distribution (these data were log-transformed before statistical analyses). *p* ≤ 0.05 was considered significant. 

With an α level of 0.05, a power established at 90% and an effect size of 0.90, the required total sample size was 32 (i.e., n = 8/group). The hypothesized effect size of 0.90 was calculated from previous studies performed using this model [42]. Two-way ANOVA calculations using the hypothesized effect size and the total sample size of 32 (i.e., n = 8/group) indicated that the actual power achieved in this study was 90.6%.

## 3. Results

The detailed composition of the ingredients of rats’ diets is shown in Table 1. In Labofeed H, LA and ALA predominated, while in SAF oil, the most prevalent were *c*9C18:1, followed by LA and C16:0 acids. In Bio-C.L.A., two main CLA isomers predominated (*c*9*t*11C18:2 and *t*10*c*12C18:2) which were present in a 1:1 ratio (97.6 mg and 99.6 mg per g of sample, respectively). All diet components contained tocopherols and cholesterol was detected only in laboratory fodder, while none of the dietary ingredients contained oxysterols. 

**Figure 2:**
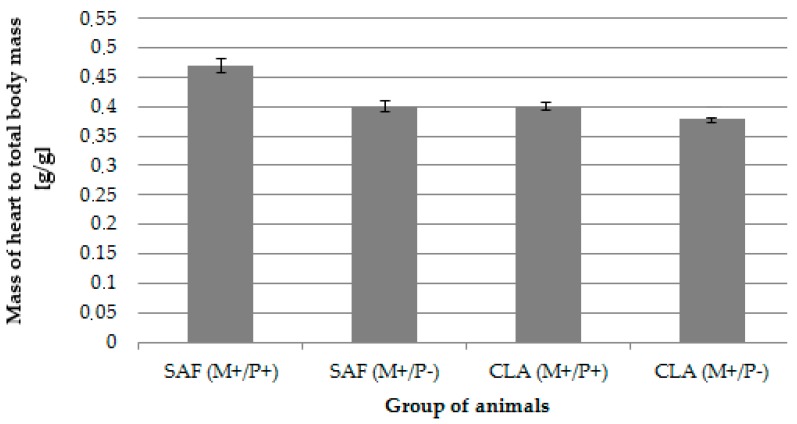
The relative mass of the hearts of offspring rats [g/g].

The supplementation of MD did not negatively influence health status, which was confirmed by the lack of any signs of welfare disorders as well as spontaneous tumors in mothers during the experiments. The effect of applied dietary modification (both maternal and postnatal) on body weight and cancer incidence among pups has been published elsewhere [41]. The relative mass of hearts (expressed in reference to the whole-body mass) was significantly influenced by MD and OS, although no interactions were observed. CLA isomers present in the maternal diet resulted in a significantly (*p* = 0.04) lower mass of hearts in their progeny in comparison to the hearts of pups whose mother received SAF oil. Similarly, offspring for whom supplementation with the same preparation as the mother was continued—(SAF(M+/P+) and CLA(M+/P+))—had significantly (*p* = 0.03) heavier hearts as compared to offspring fed only with laboratory fodder—(SAF(M+/P−) and CLA(M+/P−)) (Table 2/Figure 2). 

The supplementation of the mothers’ diet with CLA isomers significantly decreased the contents of C12:0 and C16:0 FA in the hearts of their offspring, especially those that did not receive supplementation (CLA(M+/P−)). The contents of C14:0, C15:0 and C21:0 FA were significantly increased by Bio-C.L.A. being present in maternal diets, particularly in the hearts of pups continuously supplemented with CLA isomers (CLA(M+/P+)). The continuous supplementation of the offspring’s diet significantly but oppositely influenced the content of C18:0 and C24:0 FA, as the C18:0 content in hearts was enhanced while C24:0 was reduced. Interactions of both dietary interventions occurred in the case of C14:0 and C18:0 (*p* = 0.01 and *p* = 0.04, respectively) (Table 2).

The hearts of the offspring of mothers receiving SAF oil contained significantly higher amounts of *c*15C24:1 FA (*p* < 0.01); in the case of *c*9C18:1, a similar tendency was observed (*p* = 0.05). Progeny fed only with laboratory chow had a considerable higher content of *c*11C18:1 and *c*11C20:1 FA (*p* = 0.01 and *p* < 0.01, respectively) in their hearts compared to supplemented animals. Moreover, in the case of *c*11C20:1, a significant interaction of MD and OS was observed (*p* < 0.01) (Table 2).

Applying dietary modifications in a different manner influenced PUFA contents in hearts. A mother’s diet containing Bio-C.L.A. significantly diminished the content of ALA content in the hearts of offspring (*p* = 0.03) but enhanced the level of EPA (*p* = 0.00). Lack of supplementation of progeny rats resulted in the significantly elevated cardiac levels of EPA, docosapentaenoic acid (*c*7*c*10*c*13*c*16*c*19C22:5; DPA) and n-3 PUFA (*p* < 0.01, *p* < 0.01 and *p* = 0.05, respectively). Interactions of maternal and progeny diets were established only for EPA content (Table 2).

The supplementation of MD with Bio-C.L.A. significantly decreased the total amount of atherogenic saturated FA (A-SFA) (*p* = 0.02) in the hearts of offspring. The ratio of the hypo to hypercholesterolemic FA (HH) was significantly affected by the MD (*p* = 0.03) as well as by the OS (*p* = 0.03). The lack of supplementation during the early postnatal period resulted in the distinct reduction of the Δ4-desaturase (D4D) index in the hearts of pups (*p* < 0.01) and a decrease of iso_ALA (*p* < 0.01). An interactive influence of both used nutritional factors was observed in the case of HH and iso_ALA indices (*p* = 0.03 and *p* = 0.04, respectively) (Table 2).

CLA group—group of mothers receiving conjugated linoleic acids (Bio-C.L.A.) during pregnancy and breastfeeding, SAF group—group of mothers receiving safflower oil during pregnancy and breastfeeding, SAF(M+/P+)—group of offspring receiving safflower oil during fetal life, breastfeeding and after separation from mothers, SAF(M+/P−)—group of offspring receiving safflower oil only during fetal life and breastfeeding, CLA(M+/P+)—group of offspring receiving conjugated linoleic acids (Bio-C.L.A.) during fetal life, breastfeeding and after separation from mothers, CLA(M+/P−)—group of offspring receiving conjugated linoleic acids (Bio-C.L.A.) during fetal life and breastfeeding.

The introduction of CLA isomers into MD resulted in a significantly elevated total content of CFA, with a special emphasis on groups of various CD isomers in the hearts of offspring. Continued administration to offspring with the same preparation as their mothers (SAF(M+/P+) and CLA(M+/P+)) considerably increased the levels of all isomers of CT compared to the hearts of animals fed with standard laboratory fodder. Similar dependencies were observed among the amounts of CD isomers, except for *tt*CD, whose significantly higher content was detected in hearts of pups deprived of any supplementation (*p* = 0.03). In the case of a sum of *ct/tc* and *cc* isomers of CD, as well as of two particular main CLAs, significant interactions of maternal and postnatal diets occurred (Table 3). 

The content of MDA in the hearts of offspring in whom the supplementation with the same preparation as mothers was continued (both SAF oil and Bio-C.L.A.) was significantly higher (*p* = 0.04) compared to the animals fed only laboratory fodder (Table 4). 

The administration of CLAs to mothers significantly increased the content of δ-tocopherol (*p* < 0.01), while γ-tocopherol acted oppositely (*p* = 0.04). The continuous supplementation of offspring diets resulted in the significant elevation of α- and γ- tocopherols as well as α-tocopherol acetate in examined hearts (Table 4). 

The total cholesterol content in the hearts of offspring was neither influenced by MD nor by OS. The progeny of dams fed with Bio-C.L.A. (CLA groups) had a significantly lower concentration of 7AOH and 7BOH as well as 5,6BE in their hearts as compared to animals born to mothers receiving SAF oil. Contents of 5,6BE and triol were significantly affected by OS (*p* < 0.01 and 0.05). Interactions of applied dietary interventions were observed in case of the cholesterol epoxides 5,6AE and 5,6BE (Table 4).

## 4. Discussion

Various adulthood disorders may originate from adverse conditions during fetal and neonatal life. Currently, a growing amount of evidence for the early origins of NCD supports the importance of this along with the earlier beliefs that chronic diseases are mainly associated with genetic abilities and an unhealthy lifestyle [13]. The nutritional state of the mother affects the supply of FAs to the fetus. During gestation, they pass through the placenta, while during lactation, they are secreted in milk to fulfill the requirements of newborns [52]. In the present study, maternal diet supplementation with CLAs lasted throughout pregnancy and lactation to establish if nutritional intervention during these critical periods could influence metabolic programming in offspring. It is noteworthy that it is claimed that early postnatal life is about as important for long-term health and disease as fetal life. Thus, by continuing the supplementation of newborn rats, we wanted to verify the main hypothesis of the “match–mismatch” theory, namely that similar pre and postnatal conditions may result in beneficial adaptation to long-term life conditions and hence prevent diseases [53]. The currently used animal model was previously successfully utilized by our group in experiments in which maternal diet supplementation with CLA caused a lower susceptibility to mammary tumors in female offspring as well as influencing n-3 and n-6 PUFA and their lipoxygensae (LOX) metabolites concentrations in serum [41,54]. The application of CLA isomers into “developmental programming” research in the breast cancer animal model, together with the strong emphasis on selected lipid biomarkers of CVD (being main breast cancer comorbidities), are the most important and novel aspects of this work. 

Although earlier studies have investigated the influence of CLA incorporated into maternal diet on the various metabolic dysfunctions in their offspring [55,56,57,58], little is still known regarding how the supplementation of the mother’s diet with CLA affects progeny with cancer. Similarly, the impact of maternal diet during the fetal period on heart development has hardly been described. The heart is the first organ which functions in the embryo and has either a negligible or short-term ability to regenerate after injury [59]. The results of Porrello et al. show that, after one week of postnatal life, the mouse heart loses its regenerative potential. This particular period covers the time of the critical window when cardiomyocytes are withdrawn from the cell cycle after transformation into binucleated cells [60]. This observation emphasizes the need for research aimed at the explanation of how the hearts of young organisms are influenced by various early environmental factors. Among internal organs and body structures, heart and blood vessels are by far the most affected by inadequate nutrition, but the reasons for this phenomenon remain still unknown [13]. 

To compare the impact of mothers’ and their progeny’s diet in the present study, offspring within the groups of supplementation was divided into two sub-groups: one of which was supplemented with the same preparation as mothers, and the other one was not supplemented. The quantitative composition of Bio-C.L.A. declared by the manufacturer was confirmed chromatographically by the detection of two main CLA isomers in nearly equal amounts (Table 1). These CLAs were also incorporated into the myocardium of progeny in the same ratio (1:1), regardless of whether the supplementation of offspring was continued or interrupted. The hearts of the offspring of mothers receiving Bio-C.L.A. with continuous supplementation contained more than thrice as high levels of the two main CLAs as the hearts of the pups of the same mothers in whom supplementation was stopped (Table 2). The diets supplemented with the CLA substantially decreased the weight of offspring hearts, which contrasted with the previous results of Czauderna et al. [61].

CLA isomers present in the MD significantly increased the total content of CFAs and CD in the hearts of progeny, regardless of whether their supplementation was continued (CLA(M+/P+) vs. SAF(M+/P+) and CLA(M+/P−) vs. SAF(M+/P−)). The hearts of progeny receiving the same supplementation as mothers were characterized by a higher content of CT (SAF(M+/P+) vs. SAF(M+/P−) and CLA(M+/P+) vs. CLA(M+/P−)). These results are interesting because they confirm the ability of rats (monogastric species) to isomerize FAs, as proven by the presence of not only ct/tc isomers that were delivered with the diet but also the other geometrical isomers tt and cc. Additionally, values of iso_ALA, significantly influenced by OS, confirmed this observation (Table 2). The detection of CT in the hearts of progeny indicate rats’ ability to incorporate unsaturated bonds into the FAs chain. This may result not only from the activity of endogenous enzymes but also from the activity of the microbiota inhabiting the cecum of rats. That presumption is supported by our earlier observations [62] of the result of Chaplin et al. who established the prebiotic effect of CLA [63]. 

It has been shown that the supplementation of MD affects the FA profile of the hearts of offspring during the coexisting cancer process. FA content is of special attention in terms of CVD risk, while it can exhibit different properties; e.g., SFA is considered pro-atherogenic (promotes the adhesion of lipids to the circulatory system cells) and pro-thrombogenic (stimulates clot formation in blood vessels), while unsaturated FAs (UFA) are known to include both anti-atherogenic (inhibiting the aggregation of atherosclerotic plaque and decreasing the phospholipids content) and anti-thrombogenic fatty acids [45,46]. Thus, in the present study, the total content of particular FA groups (e.g., ƩSFA, ƩMUFA, ƩPUFA, A-SFA, T-SFA) was analyzed. A negligible impact of dietary interventions in the case of most abovementioned parameters may result; e.g., from the application of the CLA isomers mixture in the present study. According to Alasnier et al. [64], the feeding of rodents with individual *t*10*c*12CLA isomers changed the FA profile of rats’ hearts to a greater extent than with *c*9*t*11CLA. The results of Kelley et al. [65] indicate an opposite effect of these two CLA isomers on FA contents in cardiac tissue. The beneficial influence of MD was considerable in the case of A-SFA. The lowest levels of C12:0, C16:0 and C18:0 being detected in hearts of pups from CLA(M+/P−) group indicates that the supplementation only of the mother’s diet with CLAs exerts sufficient a protective impact against some CVD risk biomarkers (Table 2). 

Not only the intake of particular FAs but also their mutual ratio are important CVD risk factors [31]. Thus, AI and TI were calculated on the basis of the FA profile of offspring hearts; however, applied dietary interventions did not influence their values. Taking into account the fact that UFAs may have a diversified influence on the concentration of cholesterol (hypo- or hypercholesterolemic), the relative ratios of FAs of these properties (HH) were considered as an important factor in the present study [46]. MD supplementation with CLAs as well as its continuation during the postnatal period extent increased the HH in the hearts of the CLA(M+/P+) group to the greatest (Table 2).

Another beneficial effect of continuous supplementation observed in this study was the decrease of AA content in the hearts of offspring. A reduction of the AA amount may inhibit the formation of its pro-inflammatory (prostaglandin E_2_) and pro-thrombogenic (thromboxane A_2_) metabolites arising from the cyclooxygenase pathway. AA itself is endogenously synthesized from LA as a result of desaturase and elongase activity. There is a competition between n-3 and n-6 FAs for binding sites of desaturares, elongases, cyclo- and lipooxygenases [31]. A similar competition can be observed among CLA and n-3 and n-6 PUFA, which was confirmed in hepatic microsomes in our previous studies [66]. The highest desaturase activity was found in the SAF(M+/P+) group, which proves that the addition of SAF oil, rich in LA, increases the activity of D5D and D6D. If SAF oil supplementation was carried out only for mothers (SAF(M+/P−) group), this effect was less pronounced. In the case of CLA supplementation, the observed effect was the opposite, as the two-stage diet supplementation tended to reduce the activity of D5D and D6D [66]. Values of desaturase activity indices obtained in the present study indicate the lower activity of these enzymes in cardiac than in hepatic tissue. Continuous diet supplementation increased the D4D activity in examined hearts, whereas the activity of other desaturases was comparable regardless of diet modification (Table 2). 

Cholesterol is closely related with FA metabolism [15]. The fetal requirement for cholesterol is high, because it plays an important role in the development of the growing organism. Cells require cholesterol also for proliferation and differentiation, as well as for intercellular communication. It is also a component of cell membranes and is responsible for their fluidity and permeability [30]. Cholesterol deposition in the cardiac tissue of all examined groups was similar regardless of diet modifications (Table 4). 

Cholesterol present in lipid bi-layers is largely susceptible to oxidation [67]. During this modification, many 27-carbon derivatives arise, which are more polar and thus more bioreactive than the parent compound. Oxysterols are considered either as intermediates in the catabolism of cholesterol or as bioactive lipids [68]. Under physiological conditions, the concentration of oxysterols is three orders of magnitude lower than that of cholesterol, so the impact of oxysterols on the membrane structure and properties is minor. In pathological states, the concentration of oxysterols increases, and the impact of oxysterols incorporated into intracellular membranes on their biophysical properties may be considerable [69]. Recently, a great deal of attention has been paid to the implication of oxysterols in chronic diseases with a disturbed lipid profile and oxidative stress, such as atherosclerosis. In the course of this pathology, oxysterols, by the induction of oxidative stress and inflammation, at first initiate the impairment of vascular endothelium function, and afterwards, following excessive accumulation, contribute to the formation of foam cells, plaque progression, instability and possible rupture [70]. In foam cells, oxysterols constitute 30% of the total cell sterol content [67]. Taking into account the fact that atherosclerosis concerns not only the arteries but also valves of the heart [71], as well as that the main target organs for oxysterols are tze heart and coronary vessels [67], the detection of this oxidized cholesterol derivative in cardiac tissue seem to be justified. Moreover, unlike cholesterol, oxysterols are readily able to cross lipophilic membranes, and their biological activity surpasses several times the activity of cholesterol itself [72]. The obtained results indicate that continuous offspring diet supplementation with SAF oil enhanced oxysterol formation in their hearts, which is confirmed by the highest levels of all detected oxysterols being in the SAF(M+/P+) group. 7OHs are the major oxysterols occurring in an atherosclerotic lesion, while 7K promotes the formation of foam cells and together with 7BOH is present in the atherosclerotic plaque [67]. Adachi et al., who observed increased levels of oxysterols in cardiac tissue, linked this observation with alcohol-induced increased oxidative stress and possible membrane changes [73]. Both fetal and postnatal CLA supplementation in the most effective way reduced the 7BOH accumulation in the hearts of rats, which emphasizes the beneficial effect of prolonged CLA intake on progeny cardiac health (Table 4). This also accompanies the diminished breast cancer incidence observed in the CLA-supplemented groups, as oxysterols exhibit procarcinogenic properties [67,74].

The heart is considered to be a tissue with a high rate of FA oxidation [15]. MDA, one of the markers of oxidative stress, may arise via the non-enzymatic oxidation of PUFAs or may be formed as a by-product of their enzymatic oxidation, which occurs during eicosanoid transformations. From the formation sites, MDA easily diffuses across the membranes to enter even very distant tissues, and there, through the possibility of forming covalent bonds with other molecules, it can modify their structure and properties [75]. MDA adducts by cross-linking with collagen, which may contribute to the stiffening of cardiovascular tissue [76]. By disrupting the hydrophobicity of the inner lipid layer of cell membranes and the bilayer structure of the membrane, MDA modifies the physical properties of cell membranes (e.g., polarity, fluidity, permeability) which leads to impairments in their normal functioning [77]. The results obtained in present study indicate that although prolonged supplementation increased MDA levels in cardiac tissue, its elevation was independent of the applied dietary supplement. Conjugated double bonds are believed to be more prone to oxidation; however, CLA isomers did not stimulate MDA formation, as its levels were comparable in the hearts of SAF and CLA-supplemented progeny. This was previously confirmed also in hepatic tissue [66]. 

The lipid oxidation processes are controlled by various antioxidants; e.g., tocopherols. Their content in the fetal plasma is lower than in their mothers, but increases during the pregnancy [30]. Tocopherol amounts deposited in progeny tissues depend also on the intensity of lipid oxidation. A high rate of lipid oxidation in cardiac tissue may be diminished by tocopherols (which was confirmed by the obtained results concerning MDA levels and PI). The prolonged diet supplementation of pups in general increased the tocopherol content in hearts. The most pronounced effect of CLA supplementation was observed in the case of δ-tocopherol (Table 4), although the results of Zeitz et al. [78] reported only a minimal effect of CLA on the tissue tocopherol status in pups.

## 5. Conclusions

The results of the present study emphasize the efficacy of dietary CLA supplementation during critical windows of development in maintaining long-term health status. Aside from the previously reported anticancerogenic properties, CLA also beneficially influences selected CVD lipid biomarkers in the nutritional programming animal model. Further research is required to evaluate the optimal dose, isomeric composition and safety of CLA usage in humans during pregnancy and breastfeeding to reduce the risk of non-communicable diseases. It has emerged that the verification of the novel hypothesis concerning CLA properties may contribute to the development of nutritional knowledge and, as a consequence, lead to the prevention of concomitant diseases. 

## Figures and Tables

**Table 1:** Composition of dietary ingredients.

Fatty Acid	Labofeed H	Safflower Oil (SAF Oil)	Bio-C.L.A.
**C6:0** (μg/g)	10.4	nd	nd
**C8:0** (mg/g)	nd	nd	1.23
**C10:0** (μg/g)	nd	nd	917
**C12:0** (μg/g)	4.8	nd	29.2
**C14:0** (μg/g)	18.6	209	209
**C15:0** (μg/g)	10.0	53.8	nd
**C16:0** (mg/g)	1.05	13.9	16.2
***c7* C16:1** (μg/g)	11.9	167	31.8
***c*9 C16:1** (μg/g)	16.4	266	235
**C17:0** (μg/g)	10.0	61.4	77.5
***c6*C17:1** (μg/g)	6.2	nd	nd
***c*9C17:1** (μg/g)	nd	69.0	54.2
**C18:0** (mg/g)	0.44	0.01	6.48
***t*11C18:1** (μg/g)	nd	nd	53.4
***c*9 C18:1** (mg/g)	1.12	130	37.0
***c*11 C18:1** (mg/g)	0.04	2.12	2.66
***t*9*c*12 C18:2** (μg/g)	nd	nd	202
**LA** (mg/g)	4.12	75.3	42.7
**ALA** (mg/g)	2.21	0.95	0.00
**C20:0** (μg/g)	10.5	957	820
***c*9*t*11C18:2** (mg/g)	nd	nd	99.6
***t*7*c*9C18:2** (μg/g)	nd	nd	944
***t*10*c*12C18:2** (mg/g)	nd	nd	97.6
***c*11*c*13C18:2** (mg/g)	nd	nd	4.13
***c*9*c*11C18:2** (μg/g)	nd	nd	699
***c*11C20:1** (mg/g)	10.4	619	nd
***c*8*c*11*c*14*c*17C20:4n-3** (μg/g)	nd	672	nd
**C22:0** (μg/g)	5.4	nd	359
**C24:0** (μg/g)	nd	202	64.9
***c*15 C24:1** (μg/g)	nd	240	187
**Conjugated fatty acids:** (mg/g)			
**ƩCFA:**	nd	0.49	192
**ƩCD:**	nd	0.23	189
***tt* CD**	nd	0.17	5.18
***ct/tc* CD**	nd	0.05	178
***cc* CD**	nd	nd	6.23
**ƩCT:**	0.00	0.26	3.00
***ttt* CT**	nd	0.22	2.78
***ttc* CT**	nd	0.02	0.22
***cct* CT**	0.00	0.02	0.00
**Cholesterol** (μg/g)	155	nd	nd
**Tocopherols** (μg/g)			
**δ (delta) tocopherol**	27.2	46.7	64.2
**γ (gamma) tocopherol**	4.43	18.5	21.0
**α (alpha) tocopherol**	14.7	22.3	186
**α (alpha) tocopherol acetate**	82.4	129	48.6

LA—linoleic acid; ALA—α-linolenic acid; CFA—conjugated fatty acids, CD—conjugated dienes, CT—conjugated trienes, *cc*—*cis,cis* isomers*, ct/tc*—*cis,trans/trans,cis* isomers, *tt*—*trans,trans* isomers, *ttt*—*trans,trans,trans* isomers, *ttc*—*trans,trans,cis* isomers, *cct*—*cis,cis,trans* isomers; nd—not detected.

**Table 2:** Profile of fatty acids (FA) in hearts of the offspring of mothers supplemented with safflower oil (SAF oil) or Bio-C.L.A., in whom supplementation with the same preparation was continued (SAF(M+/P+); CLA(M+/P+)) or not (SAF(M+/P−); CLA(M+/P−)).

Mothers’ Diet		SAF Oil		Bio-C.L.A.		*p* Values for Two-Way Anova		
	Group	SAF (M+/P+) (n = 10)	SAF (M+/P−) (n = 9)	CLA (M+/P+) (n = 8)	CLA (M+/P−) (n = 9)	Mothers’ Diet (MD)	Offspring Supplementation (OS)	Interaction (MD × OS)
Variables								
**Fatty acids:**								
**ƩFAs** (mg/g)		6.20 ± 0.71	7.03 ± 0.77	6.43 ± 0.63	6.14 ± 1.24	0.26	0.37	0.06
**C12:0** (μg/g)		5.84 ± 2.73	5.77 ± 2.59	5.58 ± 1.89	2.86 ± 0.75	0.04	0.07	0.09
**C14:0** (μg/g)		13.6 ± 2.92 ^a^	17.5 ± 3.43 ^ab^	20.5 ± 4.75 ^b^	16.9 ± 5.21 ^ab^	0.03	0.92	0.01
**C15:0** (μg/g)		7.84 ± 2.47	9.37 ± 1.26	10.9 ± 3.52	9.75 ± 1.93	0.04	0.82	0.10
**C16:0** (μg/g)		914 ± 53.8	941 ± 120	868 ± 63.9	821 ± 104	0.01	0.74	0.23
**C17:0** (μg/g)		37.7 ± 7.27	40.6 ± 5.71	40.1 ± 7.79	46.0 ± 13.2	0.22	0.17	0.64
**C18:0** (mg/g)		1.72 ± 0.10 ^ab^	1.71 ± 0.23 ^ab^	1.77 ± 0.13 ^b^	1.52 ± 0.20 ^a^	0.24	0.04	0.04
**C20:0** (μg/g)		7.34 ± 2.76	7.55 ± 2.87	9.10 ± 2.75	6.39 ± 2.03	0.74	0.16	0.11
**C21:0** (μg/g)		0.00 ± 0.00	0.00 ± 0.00	4.31 ± 1.07	4.08 ± 1.03	<0.01	0.65	0.65
**C22:0** (μg/g)		5.77 ± 2.96	4.33 ± 1.22	6.93 ± 1.23	3.67 ± 0.97	0.89	0.20	0.60
**C24:0** (μg/g)		15.7 ± 3.97	22.5 ± 4.27	15.4 ± 2.58	18.4 ± 3.95	0.09	<0.01	0.14
**ƩSFA** (mg/g)		2.71 ± 0.15	2.66 ± 0.24	2.63 ± 0.29	2.44 ± 0.312	0.10	0.19	0.40
**A-SFA** (μg/g)		932 ± 53.8	964 ± 123	786 ± 311	840 ± 108	0.02	0.45	0.85
**T-SFA** (mg/g)		2.47 ± 0.54	2.67 ± 0.34	2.55 ± 0.29	2.35 ± 0.31	0.37	0.97	0.14
***c*7C16:1** (μg/g)		9.84 ± 4.38	13.4 ± 4.99	14.4 ± 9.11	10.2 ± 3.75	0.75	0.88	0.06
***c*9C16:1** (μg/g)		12.9 ± 4.50	14.7 ± 3.52	12.5 ± 4.71	11.9 ± 3.19	0.24	0.68	0.37
***c*9C18:1** (μg/g)		208 ± 39.8	228 ± 38.8	189 ± 47.1	199 ± 14.1	0.05	0.19	0.81
***c*11C18:1** (μg/g)		171 ± 13.1	189 ± 17.0	162 ± 17.5	179 ± 18.9	0.16	0.01	0.91
***c*11C20:1** (μg/g)		5.43 ± 2.20 ^ab^	13.3 ± 5.32 ^b^	8.41 ± 3.11 ^ab^	0.00 ± 0.00 ^a^	0.85	<0.01	<0.01
***c*15C24:1** (μg/g)		8.25 ± 0.47	8.31 ± 3.17	0.00 ± 0.00	0.00 ± 0.00	<0.01	0.97	0.97
**ƩMUFA** (μg/g)		403 ± 47.4	433 ± 92.3	381 ± 74.4	384 ± 95.4	0.20	0.54	0.63
**LA** (mg/g)		1.71 ± 0.21	1.99 ± 0.21	1.77 ± 0.31	1.80 ± 0.16	0.42	0.50	0.13
**ALA** (μg/g)		13.8 ± 2.10	17.0 ± 2.26	13.3 ± 1.76	13.4 ± 3.71	0.03	0.06	0.09
***c*11*c*14C20:2** (μg/g)		10.2 ± 4.36	9.53 ± 3.00	7.30 ± 3.72	8.91 ± 1.98	0.11	0.67	0.29
**DGLA** (μg/g)		12.7 ± 3.17	15.4 ± 2.98	15.7 ± 3.60	14.4 ± 2.36	0.34	0.55	0.07
**AA** (mg/g)		0.96 ± 0.08	1.12 ± 0.11	0.99 ± 0.11	1.04 ± 0.18	0.54	0.02	0.23
**EPA** (μg/g)		0.00 ± 0.00 ^a^	7.70 ± 1.46 ^b^	7.29 ± 0.67 ^b^	5.69 ± 2.22 ^b^	<0.01	<0.01	<0.01
**DPA** (μg/g)		96.4 ± 19.7	126 ± 25.6	92.3 ± 16.7	121 ± 22.1	0.53	<0.01	0.96
**DHA** (μg/g)		482 ± 85.4	575 ± 92.8	526 ± 79.1	503 ± 96.9	0.64	0.24	0.06
**ƩPUFA** (mg/g)		3.28 ± 0.29	3.85 ± 0.39	3.42 ± 0.47	3.31 ± 0.86	0.28	0.21	0.07
**n-3PUFA** (μg/g)		590 ± 87.0	720 ± 108	636 ± 187	642 ± 116	0.64	0.05	0.08
**n-6PUFA** (mg/g)		2.69 ± 0.26	3.13 ± 0.29	2.77 ± 0.31	2.66 ± 0.77	0.25	0.30	0.09
**n-6/n-3**		4.62 ± 0.33	4.38 ± 0.38	4.40 ± 0.13	4.09 ± 1.04	0.30	0.27	0.90
**Indices:**								
**D4D**		0.83 ± 0.04	0.82 ± 0.02	0.85 ± 0.02	0.81 ± 0.02	0.84	<0.01	0.06
**D5D**		0.99 ± 0.01	0.99 ± 0.00	0.99 ± 0.00	0.99 ± 0.00	0.14	0.74	0.11
**D9D_C16**		0.01 ± 0.01	0.02 ± 0.00	0.02 ± 0.01	0.01 ± 0.00	0.57	0.27	0.23
**D9D_C18**		0.10 ± 0.01	0.12 ± 0.03	0.09 ± 0.02	0.10 ± 0.04	0.38	0.19	0.44
**D9D_total**		0.07 ± 0.03	0.08 ± 0.02	0.07 ± 0.02	0.07 ± 0.03	0.59	0.32	0.29
**PI**		121 ± 11.0	125 ± 4.30	121 ± 9.19	128 ± 5.21	0.57	0.05	0.77
**AI**		0.27 ± 0.03	0.24 ± 0.01	0.23 ± 0.09	0.26 ± 0.11	0.77	0.94	0.15
**TI**		0.75 ± 0.16	0.68 ± 0.04	0.74 ± 0.13	0.70 ± 0.12	0.86	0.18	0.69
**HH**		3.72 ± 0.35 ^a^	4.24 ± 0.21 ^ab^	23.09 ± 4.96 ^b^	4.09 ± 0.94 ^ab^	0.03	0.03	0.03
***iso***_**LA**		0.02 ± 0.01	0.03 ± 0.01	0.01 ± 0.01	0.14 ± 0.32	0.21	0.40	0.38
***iso***_**ALA**		0.61 ± 0.18 ^c^	0.29 ± 0.16 ^a^	0.45 ± 0.23 ^abc^	0.38 ± 0.12 ^ab^	0.52	<0.01	0.04

Data are shown as mean values ± standard deviation (SD). *p* value ≤0.05—significant differences among groups in two-way ANOVA. 0.00—amount was below the quantification limit (<LOQ). MD—mothers’ diet; OS—offspring supplementation, MD x OS—interaction; When interaction (MD x OS) occurs, the significance of differences among groups was further analyzed by post hoc HSD Tuckey test or multiple comparison tests. ^abc^—values with different superscripts in rows significantly differ at *p* value ≤0.05. CLA group—group of mothers receiving conjugated linoleic acids (Bio-C.L.A.) during pregnancy and breastfeeding, SAF group—group of mothers receiving safflower oil during pregnancy and breastfeeding, SAF(M+/P+)—group of offspring receiving safflower oil during fetal life, breastfeeding and after separation from mothers, SAF(M+/P−)—group of offspring receiving safflower oil only during fetal life and breastfeeding, CLA(M+/P+)—group of offspring receiving conjugated linoleic acids (Bio-C.L.A.) during fetal life, breastfeeding and after separation from mothers, CLA(M+/P−)—group of offspring receiving conjugated linoleic acids (Bio-C.L.A.) during fetal life and breastfeeding.

**Table 3:** Profile of conjugated fatty acids (CFA) in the hearts of offspring of mothers supplemented with safflower oil (SAF oil) or Bio-C.L.A., in whom supplementation with the same preparation was continued (SAF(M+/P+); CLA(M+/P+)) or not (SAF(M+/P−); CLA(M+/P−)).

Mothers’ Diet		SAF Oil		Bio-C.L.A.		*p* Values for Two-Way ANOVA		
	Group	SAF (M+/P+) (n = 10)	SAF (M+/P−) (n = 9)	CLA (M+/P+) (n = 8)	CLA (M+/P−) (n = 9)	Mothers’ Diet (MD)	Offspring Supplementation (OS)	Interaction (MD × OS)
Variables								
**Conjugated fatty acids:**								
**Ʃ CFAs** (μg/g)		60.4 ± 18.6	52.7 ± 14.5	100 ± 15.7	84.0 ± 28.8	<0.01	0.08	0.50
**Ʃ CD** (μg/g)		40.1 ± 14.6	44.6 ± 14.9	81.2 ± 10.5	75.1 ± 29.7	<0.01	0.90	0.40
***tt* CD** (μg/g)		35.1 ± 9.81	41.0 ± 14.1	46.5 ± 9.43	65.5 ± 26.8	0.03	0.03	0.24
***ct/tc* CD** (μg/g)		2.05 ± 1.88 ^a^	3.28 ± 1.86 ^ab^	32.9 ± 3.76 ^c^	8.05 ± 2.91 ^abc^	<0.01	<0.01	0.02
***t*10*c*12CLA** (μg/g)		0.97 ± 0.53 ^a^	1.28 ± 1.08 ^ab^	14.0 ± 1.83 ^c^	3.42 ± 1.12 ^abc^	<0.01	0.08	0.05
***c*9*t*11CLA** (μg/g)		0.67 ± 0.34 ^a^	0.76 ± 0.45 ^ab^	14.4 ± 1.99 ^c^	2.31 ± 0.61 ^abc^	<0.01	0.05	0.04
***cc* CD** (μg/g)		0.00 ± 0.00 ^a^	1.05 ± 0.64 ^ab^	1.60 ± 0.35 ^c^	0.44 ± 0.25 ^ab^	<0.01	<0.01	<0.01
**Ʃ CT** (μg/g)		20.2 ± 9.55	8.13 ± 5.85	15.4 ± 7.25	8.93 ± 5.43	0.42	<0.01	0.26
***ttt* CT** (μg/g)		8.77 ± 3.13	5.24 ± 2.79	7.03 ± 2.44	4.58 ± 1.55	0.19	<0.01	0.55
***ttc* CT** (μg/g)		3.82 ± 1.48	1.02 ± 0.88	2.51 ± 1.43	1.36 ± 1.02	0.39	<0.01	0.15
***cct* CT** (μg/g)		6.20 ± 3.56	0.66 ± 0.14	6.41 ± 4.77	2.16 ± 2.03	0.83	<0.01	0.97

Data are shown as mean values ± standard deviation (SD). *p* value ≤0.05—significant differences among groups in two-way ANOVA. 0.00—amount was below the quantification limit (<LOQ). MD—mothers’ diet; OS—offspring supplementation, MD × OS—interaction; When interaction (MD × OS) occurs, the significance of differences among groups was further analyzed by post hoc HSD Tuckey test or multiple comparison tests. ^abc^—values with different superscripts in rows significantly differ at *p* value ≤0.05. CLA group—group of mothers receiving conjugated linoleic acids (Bio-C.L.A.) during pregnancy and breastfeeding, SAF group—group of mothers receiving safflower oil during pregnancy and breastfeeding, SAF(M+/P+)—group of offspring receiving safflower oil during fetal life, breastfeeding and after separation from mothers, SAF(M+/P−)—group of offspring receiving safflower oil only during fetal life and breastfeeding, CLA(M+/P+)—group of offspring receiving conjugated linoleic acids (Bio-C.L.A.) during fetal life, breastfeeding and after separation from mothers, CLA(M+/P−)—group of offspring receiving conjugated linoleic acids (Bio-C.L.A.) during fetal life and breastfeeding. CFA—conjugated fatty acids, CD—conjugated dienes, CT—conjugated trienes, cc—cis,cis isomers, ct/tc—cis,trans/trans,cis isomers, tt—trans,trans isomers, ttt—trans,trans,trans isomers, ttc—trans,trans,cis isomers, cct—cis,cis,trans isomers.

**Table 4:** The contents of malondialdehyde (MDA), tocopherols, cholesterol and their oxidized derivatives in the hearts of offspring of mothers supplemented with safflower oil (SAF oil) or Bio-C.L.A., in whom supplementation with the same preparation was continued (SAF(M+/P+); CLA(M+/P+)) or not (SAF(M+/P−); CLA(M+/P−)).

Mothers’ Diet		SAF Oil		Bio-C.L.A.		*p* Values for Two-Way ANOVA		
	Group	SAF (M+/P+) (n = 10)	SAF (M+/P−) (n = 9)	CLA (M+/P+) (n = 8)	CLA (M+/P−) (n = 9)	Mothers’ Diet (MD)	Offspring Supplementation (OS)	Interaction (MD × OS)
Variables								
**MDA** (µg/g)		4.31 ± 0.94	3.27 ± 0.76	4.11 ± 1.30	3.83 ± 0.42	0.55	0.04	0.22
**Tocopherols:**								
**δ (delta)**		1.86 ± 1.38 ^a^	2.86 ± 1.15 ^ab^	5.74 ± 2.04 ^b^	2.90 ± 0.90 ^ab^	<0.01	0.06	<0.01
**γ (gamma)**		20.7 ± 15.8	6.13 ± 2.13	9.13 ± 2.85	4.69 ± 1.33	0.04	<0.01	0.11
**α (alpha)**		2.94 ± 3.28	0.54 ± 0.08	0.97 ± 0.32	0.65 ± 0.12	0.14	0.03	0.10
**α (alpha) acetate**		5.29 ± 2.75	2.70 ± 1.09	3.52 ± 1.04	3.30 ± 0.57	0.33	0.02	0.06
**Cholesterols and oxy-sterols:**								
**Cholesterol** (mg/g)		2.42 ± 0.80	3.07 ± 0.61	2.69 ± 1.01	2.31 ± 1.20	0.34	0.53	0.76
**7AOH** (μg/g)		2.04 ± 1.99	0.81 ± 0.30	0.73 ± 0.38	0.47 ± 0.31	0.04	0.36	0.67
**7BOH** (μg/g)		2.41 ± 2.09	2.36 ± 0.66	0.35 ± 0.03	2.00 ± 0.11	0.05	0.25	0.21
**5,6AE** (μg/g)		4.79 ± 4.75 ^b^	0.87 ± 0.01 ^a^	1.28 ± 0.11 ^b^	1.33 ± 0.38 ^ab^	0.12	0.10	0.04
**5,6BE** (μg/g)		6.23 ± 2.59 ^b^	0.69 ± 0.46 ^a^	1.79 ± 1.38 ^ab^	1.36 ± 0.57 ^ab^	0.01	<0.01	<0.01
**triol** (μg/g)		1.45 ± 1.43	0.00 ± 0.00	0.15 ± 0.02	0.00 ± 0.00	0.10	0.05	0.10
**7K** (μg/g)		3.71 ± 1.06	1.68 ± 0.18	2.01 ± 1.93	1.15 ± 0.43	0.10	0.40	0.39

Data are shown as mean values ± standard deviation (SD). *p* value ≤0.05—significant differences among groups in two-way ANOVA. 0.00—amount was below the quantification limit (<LOQ). MD—mothers’ diet; OS—offspring supplementation, MD × OS—interaction. When interaction (MD x OS) occurs, the significance of differences among groups was further analyzed by post hoc HSD Tuckey test or multiple comparison tests. ^abc^—values with different superscripts in rows significantly differ at *p* value ≤0.05. CLA group—group of mothers receiving conjugated linoleic acids (Bio-C.L.A.) during pregnancy and breastfeeding, SAF group—group of mothers receiving safflower oil during pregnancy and breastfeeding, SAF(M+/P+)—group of offspring receiving safflower oil during fetal life, breastfeeding and after separation from mothers, SAF(M+/P−)—group of offspring receiving safflower oil only during fetal life and breastfeeding, CLA(M+/P+)—group of offspring receiving conjugated linoleic acids (Bio-C.L.A.) during fetal life, breastfeeding and after separation from mothers, CLA(M+/P−)—group of offspring receiving conjugated linoleic acids (Bio-C.L.A.) during fetal life and breastfeeding. MDA—malondialdehyde; 7AOH—7α-hydroxycholesterol; 7BOH—7β-hydroxycholesterol; 5,6AE—cholesterol 5α,6α-epoxide; 5,6BE—cholesterol 5β,6β-epoxide; triol—5α-cholestane-3,5,6-triol; 7K—7-ketocholesterol.
